# A Real-Time Cell Image Segmentation Method Based on Multi-Scale Feature Fusion

**DOI:** 10.3390/bioengineering12080843

**Published:** 2025-08-05

**Authors:** Xinyuan Zhang, Yang Zhang, Zihan Li, Yujiao Song, Shuhan Chen, Zhe Mao, Zhiyong Liu, Guanglan Liao, Lei Nie

**Affiliations:** 1 School of Mechanical Engineering, Hubei University of Technology, Wuhan 430068, China; xinyuanzhang026@163.com (X.Z.); yzhangcst@hbut.edu.cn (Y.Z.); 19861837230@163.com (Z.L.); yu009497@163.com (Y.S.); 18164258504@163.com (S.C.); m2he712@163.com (Z.M.); guanglan.liao@hust.edu.cn (G.L.); 2 The State Key Laboratory of Digital Manufacturing Equipment and Technology, Huazhong University of Science and Technology, Wuhan 430074, China; zhiyong_liu@hust.edu.cn; 3 Hubei Modern Manufacturing Quality Laboratory, School of Mechanical Engineering, Hubei University of Technology, Wuhan 430068, China

**Keywords:** glioma stem cells, cell segmentation, deep learning, cell confluence, cell count, multi-scale feature fusion

## Abstract

Cell confluence and number are critical indicators for assessing cellular growth status, contributing to disease diagnosis and the development of targeted therapies. Accurate and efficient cell segmentation is essential for quantifying these indicators. However, current segmentation methodologies still encounter significant challenges in addressing multi-scale heterogeneity, poorly delineated boundaries under limited annotation, and the inherent trade-off between computational efficiency and segmentation accuracy. We propose an innovative network architecture. First, a preprocessing pipeline combining contrast-limited adaptive histogram equalization (CLAHE) and Gaussian blur is introduced to balance noise suppression and local contrast enhancement. Second, a bidirectional feature pyramid network (BiFPN) is incorporated, leveraging cross-scale feature calibration to enhance multi-scale cell recognition. Third, adaptive kernel convolution (AKConv) is developed to capture the heterogeneous spatial distribution of glioma stem cells (GSCs) through dynamic kernel deformation, improving boundary segmentation while reducing model complexity. Finally, a probability density-guided non-maximum suppression (Soft-NMS) algorithm is proposed to alleviate cell under-detection. Experimental results demonstrate that the model achieves 95.7% mAP50 (box) and 95% mAP50 (mask) on the GSCs dataset with an inference speed of 38 frames per second. Moreover, it simultaneously supports dual-modality output for cell confluence assessment and precise counting, providing a reliable automated tool for tumor microenvironment research.

## 1. Introduction

The advancement of in vitro cell culture technology has been pivotal for biomedical research, particularly in oncology [1]. Co-culturing tumor and immune cells, for instance, allows for the simulation of the complex tumor microenvironment. This provides critical insights into the molecular mechanisms of tumor heterogeneity and evolution [2,3,4] and facilitates the evaluation of novel anti-cancer drug efficacy by observing cellular responses to targeted therapies [5]. Central to such research is the quantitative analysis of cell viability, traditionally reliant on manual methods that are time-consuming, subjective, and can damage fragile cells [6]. Consequently, computer-aided diagnosis (CAD) systems have emerged as a promising alternative [7]. The core task for these CAD systems is the accurate quantification of cell growth characteristics, such as confluence and cell count. Cell confluence reflects population proliferation, and its dynamics can help identify the invasive properties of malignant tumors. Similarly, changes in cell numbers can indicate disease progression or treatment response. Therefore, real-time and precise analysis of these characteristics is crucial for accelerating disease diagnosis and targeted drug development cycles [8], which in turn necessitates efficient and accurate cellular image segmentation. However, achieving high-quality segmentation faces three significant challenges. First, optical aberrations in microscopy often degrade image quality, reducing the signal-to-noise ratio and contrast, which impairs segmentation accuracy [9]. Second, many cell types, such as glioma stem cells (GSCs), exhibit substantial multi-scale heterogeneity, with sizes ranging from 10 to 100 μm and diverse morphologies including discrete single cells and densely stacked colonies [10,11,12]. This heterogeneity complicates feature extraction. Third, many existing automated segmentation methods are computationally intensive, limiting their application in real-time analysis [13]. Therefore, there is a clear need for a segmentation method that is both precise and efficient enough to address the challenges of multi-scale heterogeneity and real-time processing in cellular imaging.

Currently, mainstream cell segmentation methodologies predominantly fall into two categories: conventional techniques and deep-learning-based frameworks [14,15,16,17,18]. Conventional approaches, which include methods like thresholding, edge detection, and region growing, each present distinct advantages and limitations. Thresholding, for instance, separates the target from the background based on a grayscale value. While computationally efficient and simple to implement, it is generally ineffective for segmenting adhered cells [19]. Edge detection methods identify cell contours by capturing abrupt grayscale changes. Although capable of preserving fine structural details, their performance is highly sensitive to parameter tuning, making them susceptible to error [20]. Similarly, region-growing methods expand from seed points to delineate adjacent cells. They can be effective for cell clusters but are prone to over-segmentation due to their sensitivity to variations in brightness [21].

Deep learning, with its strong feature extraction and end-to-end learning capabilities, overcomes the disadvantages of parameter sensitivity and limited ability of boundary handling in traditional methods, achieving notable success in cell segmentation [22,23,24]. These deep-learning-based methods are broadly classified into two-stage and single-stage frameworks. Two-stage frameworks, exemplified by Mask R-CNN [25], enhance feature extraction using mechanisms like Region of Interest (RoI) alignment [26,27,28,29]. However, their computational intensity significantly constrains real-time performance, motivating extensive research into more efficient single-stage architectures. Among single-stage methods, U-Net [30] and its derivatives have achieved remarkable success, particularly in segmenting fluorescently labeled cells, thanks to its symmetric encoder–decoder structure and skip connections. Subsequent models like CP-Net [31], HoLy-Net [32], and YeastNet [33] have integrated attention mechanisms and hybrid loss functions to better distinguish overlapping cells. Nevertheless, these models often struggle with blurred boundaries and feature confusion in dense regions, particularly when applied to unprocessed bright-field images. To better balance accuracy and efficiency, the you only look once (YOLO) series introduced a new paradigm for single-stage segmentation. YOLOv8, a prominent model in this series, leverages a cross-scale feature fusion mechanism that combines high-level semantic information with low-level details. This enhances the recognition of cell boundaries and improves inference speed [34]. Due to these strengths, we adopt YOLOv8 as the baseline model for this study. Despite these advancements, YOLOv8 faces two critical challenges. First, the traditional fixed-structure feature pyramid network (FPN) exhibits cross-scale feature matching deviations in multimodal cell images [35]. In practice, cell morphologies vary greatly, and traditional feature fusion struggles to balance microscopic textures with macroscopic shapes, causing missed small-cell detections and reduced boundary accuracy. Second, a prominent contradiction exists between the need for model lightweighting and the quadratic growth in convolutional parameters. To overcome these two challenges, NAS-FPN [36] has been employed to optimize the feature interaction path through neural network architecture search, thereby improving the efficiency of feature fusion. However, the irregular connection structure generated by NAS-FPN has led to an expansion of model parameters, which poses difficulties for deployment in lightweight environments. Similarly, while ASF-YOLO [37] has shown promise on public datasets using techniques like depth-wise separable convolution, its performance on unprocessed bright-field cell images has not been thoroughly evaluated.

**Figure 1:**
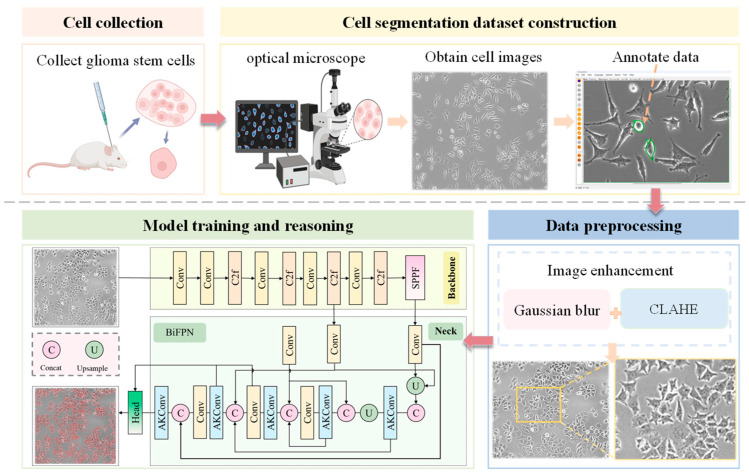
Schematic diagram of computer-aided cell image segmentation workflow, comprising data collection, dataset construction, data preprocessing, model training, and reasoning.

Based on the above analysis, two critical issues must be addressed to ensure accurate and efficient cell segmentation. First, feature extraction under complex scenarios and the multi-scale heterogeneity of cell structures require resolution. It can be addressed by reconfiguring the feature interaction pathways in FPNs. Second, the computational burden potentially introduced by improvement strategies should be minimized. To solve the second problem, morphology-adaptive convolutional kernels with dynamic receptive field regulation mechanisms could be constructed so as to alleviate the parameter explosion caused by fixed-shape convolutional kernels in traditional convolutional neural networks. Consequently, a framework based on AKConv-BiFPN (AKB)-YOLO is proposed for GSC segmentation in this study. The main contributions of this work are summarized as follows (Figure 1):

(1) An architecture is proposed to enable accurate and efficient cell segmentation. To overcome the inherent limitations of traditional FPNs in extracting multi-scale cellular features, this paper introduces a bidirectional feature pyramid network (BiFPN) with weighted fusion, which enhances the ability to extract multi-scale features through a bidirectional feature fusion strategy. Additionally, to mitigate the increased computational cost from the model improvements, adaptive kernel convolution (AKConv) is integrated into the convolutional layers. By incorporating learnable kernel offsets, this approach reduces parameter complexity while enhancing cell feature extraction ability. Through accurate and efficient cell segmentation, real-time and synchronized calculation of cell confluence and count is achieved, providing a powerful new tool for analyzing GSC growth dynamics.
(2) The model was evaluated on both our GSCs dataset and DSB2018. Experimental results show superior segmentation accuracy and inference speed on GSCs, achieving optimal precision-efficiency balance. The public dataset tests confirm excellent generalization.
(3) Glioma tissues were excised from murine brains and enzymatically digested to yield viable single-cell suspensions. From these suspensions, GSC-enriched adherent cultures were subsequently established under optimized media and culture conditions. Time-series images of the GSC cultures were then captured using an inverted fluorescence microscope to monitor morphological changes. Prior to model training, these images were preprocessed with a pipeline of Contrast-Limited Adaptive Histogram Equalization (CLAHE) and Gaussian blurring to enhance image quality.

## 2. Materials and Methods

### 2.1. Data Collection and Dataset Construction

In biomedical research, the quantity and quality of datasets are critical determinants of deep-learning model performance. This study employed a serum-free neurosphere culture system to isolate GSCs, which exhibit sustained self-renewal capacity, multipotent differentiation potential, and marked tumorigenicity, thereby recapitulating tumor heterogeneity and treatment resistance.

The cell culture protocol comprised the following steps:

Step 1: Removal of 8 mL of original medium (containing 1 mL of cell suspension);

Step 2: Addition of 2 mL of trypsin–EDTA solution (4 min digestion for endothelial cells vs. 2 min for glioma cells), exploiting trypsin’s specificity for lysine/arginine carboxyl-terminal peptide bonds;

Step 3: Neutralization with serum-containing complete medium (utilizing α1-antitrypsin inhibition);

Step 4: Centrifugation (1000 rpm, 3–4 min);

Step 5: Resuspension in fresh medium at 1:4 dilution (final volume: 8 mL). Strict aseptic techniques prevented membrane damage or apoptosis.

To obtain high-quality images for clear visualization of cell boundaries and morphology, a temperature-controlled culture device equipped with an optical microscope imaging system (CellAnalyzer, Lianhua Intelligent Manufacturing Wuhan Biotechnology Co., Ltd., Wuhan, China) was used. Cell samples were observed under a 20× objective lens over an extended period. Time-lapse imaging was performed at 10 min intervals for a total duration of 4000 min, resulting in the acquisition of 400 high-resolution time-series images, each with a resolution of 2048 × 2448 pixels. The dataset was subsequently divided into a training set and a test set at an 8:2 ratio. Cells in both sets were manually annotated using the X-AnyLabeling toolbox to delineate cell contours. All annotations were independently reviewed and verified by a senior physician with seven years of experience.

### 2.2. GSCs Image Preprocessing

After constructing the cell image analysis dataset, data preprocessing is crucial. Optical microscope-acquired cell images often exhibit high noise, low contrast, and boundary artifacts, all of which can negatively impact subsequent cell segmentation tasks. In this study, prior to inputting the data into the neural network for training, a preprocessing pipeline combining CLAHE and Gaussian filtering was implemented.

The pipeline’s first component, CLAHE, is an advanced form of histogram equalization designed to enhance image contrast while preserving local detail. It operates by dividing the image into non-overlapping sub-regions and applying equalization independently to each, thereby preventing the feature loss commonly associated with global methods. Furthermore, its contrast-limiting threshold prevents the over-amplification of noise in localized areas. The second component, Gaussian filtering, then suppresses noise by applying a weighted average to neighboring pixels based on a Gaussian function. This process effectively smooths the image while retaining critical details such as cell boundaries and morphological features.

**Figure 2:**
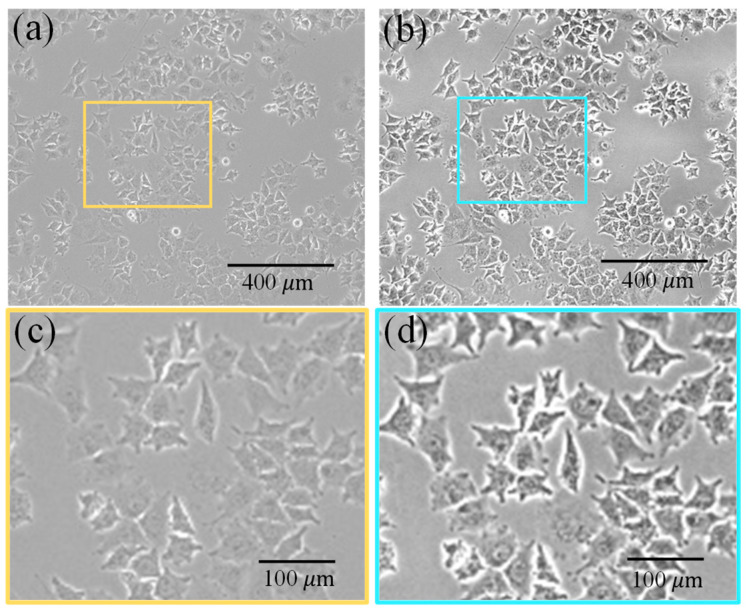
Image enhancement comparison: (**a**) Original image, (**b**) Enhanced result, (**c**) Original image detail, (**d**) Enhanced image detail.

Figure 2 shows that the preprocessing method combining CLAHE and Gaussian filtering can synergistically leverage the advantages of both techniques. This strategy significantly enhances image contrast and reduces noise while avoiding artifacts such as blurred boundaries or uneven enhancement that can arise from using a single technique. The resulting images exhibit substantially improved visual clarity and detail, providing a high-quality dataset for subsequent segmentation and feature extraction tasks.

### 2.3. The Overview of Methods

Glioma is the most common malignant tumor of the central nervous system, accounting for approximately 45% of intracranial tumors, and it is characterized by high heterogeneity, rapid proliferation, and extensive invasion [38,39,40]. When the tumor compresses or infiltrates surrounding brain tissue, it can cause severe symptoms such as epilepsy, limb paralysis, blindness, and cognitive impairment, leading to high mortality and disability rates. These rates severely threaten human health [41,42]. GSCs are considered key drivers of tumor recurrence, invasiveness, and chemoresistance. Therefore, understanding their behavior is critical. Cell segmentation technology provides a powerful means to quantify the dynamic changes in cell confluence and number during GSC division and differentiation. The goal is to elucidate the spatiotemporal dynamics of these cells, which could fundamentally alter treatment paradigms for glioblastoma and help overcome current therapeutic limitations [43,44,45,46].

The dataset in this study consists of GSC images acquired via optical microscopy. The objective is to enable precise and real-time segmentation of these cell images. GSCs are endowed with unique biological characteristics. They are usually in a spherical or near-spherical shape and exist as tumor spheres that grow in suspension, which is similar to neural stem cells. However, certain subpopulations exhibit irregular morphologies, which complicates the task of accurately identifying cell boundaries using segmentation algorithms.

**Figure 3:**
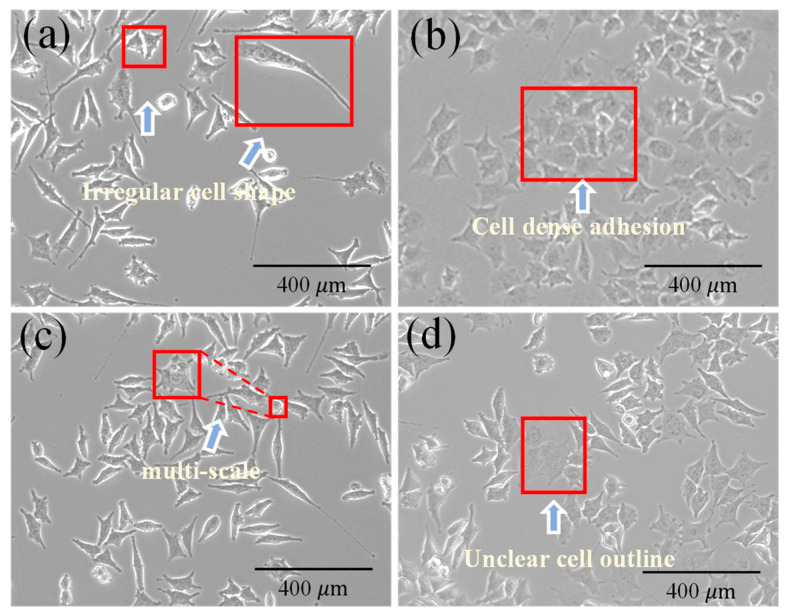
Schematic illustration of the challenges in GSC segmentation: (**a**) Irregular cell shape; (**b**) Cell dense adhesion; (**c**) Cell multi-scale; (**d**) Unclear cell outline.

Figure 3 demonstrates several common challenges encountered during the segmentation of GSCs, including irregular cell shapes (Figure 3a), dense adhesion of cells (Figure 3b), multi-scale cells (Figure 3c), and unclear cell boundaries (Figure 3d), which are shown. Moreover, due to the large size of the images and high density of cell labels, model training complexity is markedly increased, which in turn affects the feasibility of real-time biomedical device applications.

**Figure 4:**
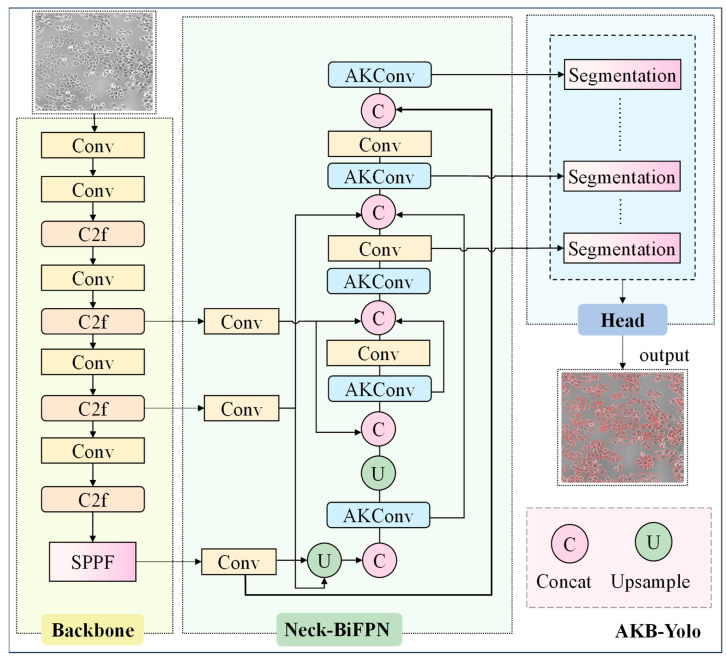
The structural diagram of the model in this study.

In the initial phase of this study, YOLOv8s-seg was selected as the base model. However, its substantial parameter size (11,780 KB) and insufficient inference speed precluded its use for real-time analysis. To address these limitations, we propose AKB-YOLO, a lightweight, single-stage segmentation framework designed to balance accuracy with computational efficiency. Specifically, considering the substantial multi-scale heterogeneity among GSCs, the original c2f layer in the neck network was replaced with AKConv [47]. AKConv dynamically adjusts convolutional kernels to better adapt to various positional and size-related cellular features in GSC images. This modification ensures segmentation accuracy while improving computational efficiency. Furthermore, to enhance feature fusion capabilities, a BiFPN was integrated into the neck network [48]. BiFPN designs efficient feature fusion pathways to rapidly achieve multi-scale feature integration and enable information exchange across different hierarchical features without notably increasing model complexity. Figure 4 illustrates the proposed AKB-YOLO network architecture.

### 2.4. Lightweight Improvement Based on AKConv Module

For cell confluence assessment and counting, model computational complexity is a critical consideration. Traditional convolutional filters are limited to extracting local features within a fixed receptive field and cannot capture information from distant locations. Moreover, traditional convolution kernels are usually fixed in a square shape. As the kernel size increases, the number of parameters grows quadratically, resulting in larger models that slow down detection. Regarding the GSCs, they have diverse shapes and sizes. The fixed sampling shape and square convolution kernel struggle to flexibly adapt to the cell features. Thus, the AKConv module is employed to replace traditional convolution. AKConv overcomes the limitations of fixed-shape kernels by supporting arbitrary sampling shapes, both symmetric and asymmetric, and a flexible number of parameters, thereby providing greater design freedom for the network. Through offset learning and resampling, AKConv realizes a dynamically deformable convolutional kernel that adapts to complex target shapes. This design is especially beneficial for cell images with blurred boundaries, overlapping cells, or heterogeneous morphologies, enhancing the model’s ability to capture subtle features. The module dynamically adjusts its sampling shape to better conform to irregular cell contours and reduce background interference. In addition, the ability to linearly scale the number of parameters addresses the computational requirements of processing high-resolution medical images, reducing parameter count while maintaining high accuracy. In our study, the AKConv module was configured with num_param = 3, stride = 1, and bias = False, utilizing Batch Normalization (BN) and the SiLU activation function.

**Figure 5:**
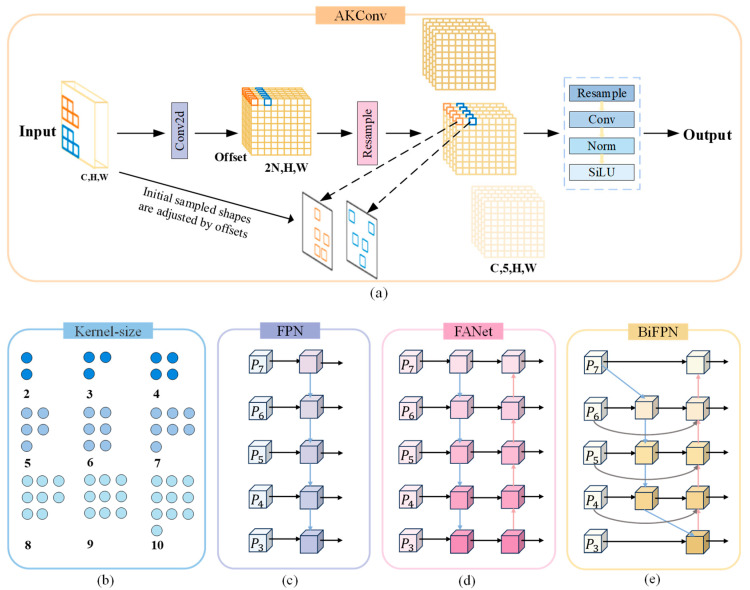
Module structure diagram. (**a**) The detailed schematic of the structure of AKConv; (**b**) The initial sampled coordinates for arbitrary convolutional kernel sizes; (**c**) FPN network architecture; (**d**) PANet adds an additional bottom-up pathway on top of FPN; (**e**) BiFPN structure is based on PAN and uses the fast normalized fusion method for fusion with weights with better accuracy and efficiency trade-offs.

As shown in Figure 5a, consider the AKConv module with a convolution kernel size of 5. A coordinate generation algorithm based on grid expansion is used to construct the initial sampling coordinate set ${\{P}_{n}\}$. Unlike traditional convolution, which uses a fixed center point, AKConv uses the top-left corner (0,0) as the sampling origin, supporting the initialization of convolution kernels of arbitrary shapes. This sampling method can better fit the morphological features of different cells. Next, the corresponding kernel offsets are obtained through convolution operations, with a dimension of (*B*, 2*N*, *H*, *W*), where *N* is the size of the convolution kernel, and *C*, *H*, and *W* represent the number of channels, image height, and image width, respectively. Then, the offsets are point-wise added to the original coordinates $\left(P_{0}+P_{n}\right)$ to obtain the adjusted sampling coordinates, enabling the shape of the convolution kernel to dynamically adapt to the features in cell image and accurately capture key features such as cell edge textures. The feature map is subsequently resampled according to the adjusted coordinates. After resampling, the feature map undergoes reshaping, another convolution operation, standardization, and, finally, the output of the final result through the activation function SiLU. Compared to the initial sampling configuration, the adjusted sampling configuration varies spatially, making the convolutional network more efficient and flexible in extracting features from cell images.

### 2.5. Using BiFPN Module in Neck Network

The Neck layer primarily adopts the PAN (Figure 5d) + FPN (Figure 5c) structure for feature fusion, with only one-way feature transfer. This limitation may lead to the loss of key details in the deep network. Regarding the multi-scale challenge posed by the varying cell sizes, it does not fully leverage the correlations among all pyramid feature maps. To address this, the present study introduces BiFPN into the Neck layer. Through bidirectional cross-scale connections and weighted feature fusion, BiFPN allows features to flow freely between different levels, effectively capturing multi-scale features, thereby improving the performance of the object detection task.

Figure 5e illustrates the feature fusion method of BiFPN. Compared to the traditional FPN, BiFPN introduces additional nodes between the original input and output nodes at the same level, forming a bidirectional network and removing the unidirectional information flow limitation. This enables the effective integration of multi-scale features without notably increasing the computational cost. Additionally, BiFPN allows features to flow freely between different levels and removes nodes with only one input edge (such as the first node on the right side of P7 in Figure 5e). Moreover, BiFPN introduces a weighted fusion mechanism and adopts a fast normalization fusion method, as shown in Equation (1). BiFPN adjusts the contribution degree of each layer through a set of learnable, normalized weight parameters $w_{i}$, scales the weights to the range of 0~1, and adds weights to the input features in each cell image to optimize the feature fusion process. This enables the network to prioritize features with more information content and better focus on recognizing cell boundary features.

(1) $$O=\sum_{i}\;\frac{w_{i}}{\epsilon +\sum_{j}\;w_{j}}\cdot F_{i}$$

where O denotes the fused feature, $F_{i}$ represents the input feature of the *i*-th layer, $w_{i}$ are learnable weights, and ϵ is a small constant to prevent division by zero.

BiFPN incorporates bidirectional cross-scale connections and fast normalization. Equation (2) describes the two fused features at level 6. $P_{6}^{td}$ is the intermediate feature at level 6 on the top–down path, and $P_{6}^{out}$ is the output feature at level 6 on the bottom-up path. Conv represents the depth-wise separable convolution, and batch normalization and an activation function are added after each convolution. All other features are constructed in a similar manner.

(2) $$P_{6}^{td}=Conv\left(\frac{w_{1}\times P_{6}^{in}+w_{2}\times Resize\left(P_{7}^{in}\right)}{w_{1}+w_{2}+\varepsilon }\right)\;P_{6}^{out}=Conv\left(\frac{w_{1}\times P_{6}^{in}+w_{2}\times P_{6}^{id}+w_{3}\times Resize\left(P_{5}^{out}\right)}{t_{1}+t_{2}+t_{3}+\varepsilon }\right)$$

where $P_{6}^{in}$ represents the input of the sixth layer, with corresponding weight $w_{1}$; $P_{7}^{in}$ denotes the input of the seventh layer, with associated weight $w_{2}$; and $t_{1}$, $t_{2}$, and t_3_ correspond to the weights of intermediate layers.

### 2.6. Anchor Box Optimization

Non-maximum suppression (NMS) is mainly used to eliminate redundant detection boxes. Its purpose is to ensure that each target is detected only once, thus enhancing the prediction performance of the model. The traditional NMS algorithm sorts the detection boxes according to their confidence scores. Then, the detection box with the highest confidence score is selected as the reference. The intersection over union (IoU) between this reference detection box and other detection boxes is calculated, and the detection boxes whose IoU exceeds the preset threshold are removed. In cell images, cell clusters often exist in a highly overlapping and densely distributed state. In such a situation, nearby detection boxes may be incorrectly identified as redundant and deleted by the traditional NMS algorithm. This operation directly causes the problem of missed cell detections, which seriously affects the accuracy and precision of cell segmentation.

To overcome the limitations of the traditional NMS algorithm in cell image segmentation, the Soft-NMS algorithm is introduced in this study. In this algorithm, an attenuation function for adjacent detection boxes is set based on the size of the overlapping area, instead of simply setting their scores to zero. Specifically, when a detection box has a significant overlap with the reference detection box M, its score will be greatly attenuated, reducing its probability of being selected in the subsequent screening process. On the contrary, when a detection box has only a small overlap with the reference detection box M, its original detection score will not be markedly affected, and it can still maintain a certain competitiveness in the screening process. In this way, the Soft-NMS algorithm can effectively avoid the problem of missed cell detections while eliminating redundant detection boxes, and the precision and robustness of cell segmentation are improved. The principle of the algorithm is explained by Equation (3).

(3) $$S_{i}=\left\{\begin{array}{l} S_{i},IoU\left(M,b_{i}\right)<N_{t}\\ S_{i}f\left(IoU\left(M,b_{i}\right)\right),IoU\left(M,b_{i}\right)\geq N_{t} \end{array},i=1,2,3,\ldots ,N\right.$$

where f uses a Gaussian function. $S_{i}$ denotes the confidence of the i-th detection box, $b_{i}$ represents the target detection box, M represents the detection box with the highest confidence, $IoU\left(M,b_{i}\right)$ indicates the overlap degree between M and $b_{i}$, and $N_{t}$ is the prediction threshold.

### 2.7. Loss Functions

Total loss is a weighted sum of Classification Loss (Varifocal Loss, VFL), Bounding Box Regression Loss (CIoU + DFL) and Segmentation Loss (Binary Cross-Entropy, BCE). The calculation formulas for the three loss functions are as follows:

(4) $$L_{Cls}=-\frac{1}{N}\sum_{i=1}^{N}\sum_{c=1}^{C}y_{i,c}log{\hat{y}}_{i,c}$$

where C denotes the number of categories. $y_{i,c}$ represents the ground-truth label of sample *i* for category *c*, where it is set to 1 if the sample belongs to category *c*, and 0 otherwise. ${\hat{y}}_{i,c}$ denotes the predicted probability of sample *i* belonging to category c.

(5) $$L_{\mathrm{Box}}=\lambda_{\mathrm{cor}}\sum_{i=0}^{N}\left[{\left(x_{i}-{\hat{x}}_{i}\right)}^{2}+{\left(y_{i}-{\hat{y}}_{i}\right)}^{2}+{\left(\omega_{i}-{\hat{\omega }}_{i}\right)}^{2}+{\left(h_{i}-{\hat{h}}_{i}\right)}^{2}\right]$$

where *N* denotes the number of bounding boxes, $\left(x_{i},y_{i}\right)$ represents the center coordinates of the ground-truth box, and (${\hat{x}}_{i},{\hat{y}}_{i}$) denotes the center coordinates of the predicted box. $\omega_{i}$ and $h_{i}$ correspond to the width and height of the ground-truth box, while ${\hat{\omega }}_{i}$ and ${\hat{h}}_{i}$ represent the width and height of the predicted box. $\lambda_{\mathrm{cor}}$ is a hyperparameter that controls the weight of the coordinate loss.

(6) $$L_{Seg}=-\frac{1}{N}\sum_{i=1}^{N}\left[y_{i}log{\hat{y}}_{i}+\left(1-y_{i}\right)log\left(1-{\hat{y}}_{i}\right)\right]$$

where $y_{i}$ denotes the ground-truth label of the sample, representing the true class of a pixel or region, while ${\hat{y}}_{i}$ denotes the predicted probability produced by the model.

## 3. Experiment and Result

### 3.1. Experimental Settings

The experiments were conducted on the Ubuntu 24.04.1 LTS system with an NVIDIA GeForce 3060 GPU (12GB), leveraging dependencies, including PyTorch 2.4.1, Python 3.10, and CUDA 12.4. The Stochastic Gradient Descent (SGD) optimizer was employed for model training. The training parameters are detailed in Table 1:

### 3.2. Evaluation Metrics

For the algorithm model proposed in this paper, we used several key evaluation indicators, including precision, recall rate, and mean average precision (0.5), to evaluate the model accuracy, as well as the number of parameters, floating-point operations per second (FLOPs), frames per second (FPS), and model size, to evaluate the performance of model complexity and inference speed so as to verify the performance of the method proposed in this paper on the dataset.

Model precision refers to the proportion of actual positive samples among the samples predicted as positive by the model, indicating the accuracy of the model’s prediction for positive samples. The calculation equation is as follows:

(7) $$Precision=\frac{TP}{TP+FP}$$

where true positive (TP) represents the number of correctly predicted positive samples among all samples predicted as positive, while false positive (FP) denotes the number of incorrectly predicted positive samples among all samples predicted as positive.

Recall rate refers to the proportion of true positive samples that are correctly predicted by the model among all true positive samples, representing the model’s capability to identify positive instances. This metric is calculated using the following equation:

(8) $$Recall=\frac{TP}{TP+FN}$$

where false negative (FN) represents the number of samples that are incorrectly predicted as negative but actually belong to the positive class.

Mean average precision (*mAP*) is an important metric for measuring the segmentation accuracy of a model. It is calculated as the average of all average precisions (*AP*). In segmentation tasks, mAP(box) and mAP(seg) represent the prediction accuracies of detection boxes and segmentation masks, respectively. mAP50 is the average calculated precision when the *IoU* threshold is 0.5. Specifically, for each category, the precision–recall curve is first calculated based on the *IoU* between the predicted boxes and the ground truth boxes. Then, the area under the curve is calculated, and the average of the areas for all categories is taken. *mAP* is calculated by the following equation:

(9) $$mAP=\frac{1}{n}\sum_{i=1}^{n}AP_{i}$$

where AP refers to the area encompassed by the coordinates of precision and recall, representing the average precision for each category within the samples. Here, n denotes the total number of categories in all samples.

(10) $$IoU=\frac{\left|A\cap B\right|}{\left|A\cup B\right|}$$

where A represents the predicted cell segmentation region, B denotes the ground truth cell region, and $\left|\cdot \right|$ indicates the area of a region

FLOPs represent the number of floating-point operations during the forward propagation or inference process of a model and are an important indicator for measuring the computational complexity and operational efficiency of a network.

Param (M) represents the number of parameters that the model needs to train, which mainly measures the complexity of the model. The larger the number of parameters, the more complex the model.

Weight (MB) represents the storage space size occupied by the model’s weights. The smaller the space occupied by the model, the faster the model loading speed and the more efficient the inference.

### 3.3. Ablation Study Results

A series of ablation experiments was conducted to validate the proposed method’s performance in high-density GSCs segmentation. As demonstrated in Table 2, the implementation of Soft-NMS effectively addresses missed detections in occluded cellular regions through dynamic confidence adjustment of overlapping bounding boxes, resulting in a 1.1% improvement in box mAP50 (93.4%) with only 1.8% additional parameters. Subsequent incorporation of BiFPN enhances semantic information propagation through bidirectional connections, further increasing mask mAP50 by 1.4% while maintaining computational efficiency. The replacement of conventional C2f modules with AKConv yields additional improvements, where dynamically adjusted sampling shapes enable more precise delineation of cellular boundaries and internal structures. This modification achieves a 0.6% mask mAP50 enhancement alongside a 13.9% parameter reduction, demonstrating simultaneous improvements in both accuracy and inference speed. The computational efficiency is attributed to AKConv’s linear parameter scaling characteristics, which effectively reduce resource consumption without compromising performance.

The influence of BiFPN layer depth on model performance is systematically evaluated in Table 3. Experimental results demonstrate that segmentation accuracy is maximized using a five-layer BiFPN configuration, highlighting the appreciable impact of layer depth on feature fusion capability. This optimal performance is attributed to the mechanism of dynamic multi-scale feature adjustment through learnable weights, which is enhanced by additional optimization iterations in deeper architectures. Compared to shallower three-layer and four-layer variants, the five-layer BiFPN facilitates more comprehensive contextual integration across scales, particularly improving boundary delineation and texture detail prediction in cellular segmentation. Such multi-level feature recalibration proves especially effective for capturing complex morphological variations characteristic of biomedical imaging applications.

The impact of AKConv placement on model performance is demonstrated in Table 4, where its implementation is evaluated in the backbone, neck, and both components. Optimal accuracy and inference speed are achieved when AKConv is exclusively employed in the neck, owing to its primary role in multi-scale feature fusion and enhancement. The deformable properties of AKConv are particularly effective in the neck layer, where geometric transformations between features of different scales can be better accommodated. Compared to the backbone, which is primarily responsible for computationally intensive low-level feature extraction, the neck exhibits moderate computational complexity, resulting in an optimal precision–speed balance.

### 3.4. Comparison Experimental Results

#### 3.4.1. Quantitative Analysis Results

To verify the excellent segmentation ability of the model in this paper within the same series, and considering the real-time analysis requirements for cell images in practical applications, it is required to minimize the model parameters and complexity. Therefore, some typical segmentation models with relatively small sizes in the YOLO series are selected, including YOLOv3tiny-seg, YOLOv5s-seg, YOLOv5n-seg, YOLOv8s-seg, YOLOv8n-seg, YOLOv10n-seg, YOLOv10s-seg, and the latest model in the series, YOLOv11s-seg. Table 5 shows that YOLOv8-seg demonstrates the highest precision among the models in the same series. Specifically, among the models with the size of “s”, mAP50 of the detection box and mask of YOLOv8s-seg reach 0.923 and 0.917, respectively. Compared with YOLOv5 of the same size, they are increased by 1.6% and 2.9%, respectively, and compared with the latest YOLOv11, they are increased by 0.4% and 0.6%, respectively. Although the number of model parameters and the floating-point numbers are increased, the increase in range is not significant. Therefore, YOLOv8s is selected as the base model. On this basis, the improved AKB-YOLO model has increased the mAP50 of the detection box and mask by 3.4% and 3.3%, respectively. At the same time, due to the introduction of the lightweight convolution AKConv, the model parameters are reduced by 1416 kb, and the floating-point numbers are reduced by 1 G. While improving the accuracy, the complexity of the model and the inference speed are reduced, showing excellent segmentation ability.

To assess the statistical significance of the performance improvement, we conducted a paired t-test on the mask_mAP50 values obtained from four repeated experiments. The optimized model achieved scores of 0.950, 0.946, 0.944, and 0.951, while the baseline model scored 0.917, 0.919, 0.913, and 0.915. The test yielded a t-value of 16.82 and a *p*-value of 0.00046, indicating a statistically significant improvement (*p* < 0.001) in the segmentation performance of the optimized model.

This study validates the effectiveness of the proposed method by comparing seven mainstream instance segmentation models (including benchmark models such as Mask R-CNN, ConvNeXt-V2 [49], and YOLOv8s-seg). As shown in Table 6, when the parameter scale of the proposed method is only 87.9% of that of YOLOv8s-seg, it achieves multiple performance breakthroughs. In the object detection dimension, the mAP50 of box prediction reaches 0.957, which is 3.4% higher than that of ASF-YOLO. In the instance segmentation dimension, the mask mAP50 reaches 0.95, which is 3.3% higher than that of the second-best model.

The performance improvement stems from three core innovations. First, the BiFPN cross-scale feature fusion mechanism enhances the ability to detect small objects, which is verified by the 38.6% relative improvement of the Mask index compared to SOLOv2 [50]. Second, the dynamic kernel parameter adaptation strategy of AKConv effectively expands the receptive field and shows advantages in the multi-scale representation of complex cell images. Compared with YOLACT [51], which has a similar parameter scale, the proposed method achieves absolute improvements of 36.5% and 48.6% in the mAP50 index, respectively, verifying the effectiveness of the model architecture optimization. The proposed framework establishes an accurate and computationally efficient paradigm for critical biomedical imaging applications, including histopathological analysis and in vivo microscopic imaging.

#### 3.4.2. Qualitative Analysis Result

**Figure 6:**
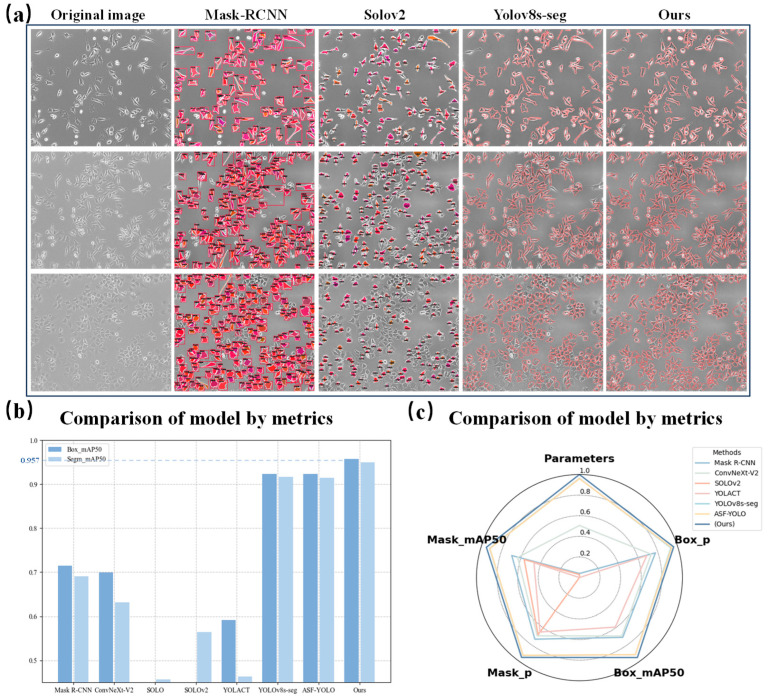
GSC cell segmentation results. (**a**) Comparison of segmentation results between the proposed method, Mask R-CNN, SOLOv2, and YOLOv8s-seg; (**b**) Bar chart comparison of eight deep-learning models using two evaluation metrics; (**c**) Radar chart comparison of eight deep-learning models using two evaluation metrics.

Figure 6a presents comparative qualitative results of glioma stem cell segmentation. Mask R-CNN exhibits jagged edge artifacts due to its fixed RoI Align sampling grid, along with marked miss-detection of small targets. SOLOv2 suffers from cell adhesion in dense regions (e.g., tumor spheroid clusters) and high miss-detection rates. YOLOv8s-seg demonstrates contour discontinuities from conventional feature pyramid limitations and poor performance in dense cellular areas. As can be seen from the figure, our method achieves superior small-target recall and more precise boundary segmentation. This benefits from the BiFPN multi-scale feature fusion mechanism, which can integrate semantic information at different levels and enhance the gradient response of cell boundaries. Meanwhile, the dynamic deformable convolution kernel of AKConv can adaptively match the irregular shapes of GSCs. These results demonstrate our model’s strong performance in segmenting highly dense, overlapping, and irregularly shaped cellular structures.

### 3.5. Analysis of Results on Public Dataset

To evaluate the cross-domain adaptation ability of the proposed model in the system, this study further conducts extended experiments on the benchmark dataset. This dataset is composed of the public dataset from the 2018 data science bowl (DSB2018) [37] competition released by the Kaggle platform, containing 670 multi-modal cell microscopic images annotated by pathologists. The dataset exhibits significant heterogeneity characteristics:

(1) The imaging parameters cover multi-level objective magnification and dual-mode imaging of fluorescence and bright field.
(2) The biological samples include heterogeneous biological tissue samples such as human hepatocellular carcinoma cells (HepG2), mouse fibroblasts (3T3), and Drosophila embryo tissues.
(3) The culture conditions involve complex environment simulations such as normoxia/hypoxia, different pH values, and metabolic states.

In the experiment, a stratified random sampling strategy is adopted to divide the dataset into a training set (n = 536) and a test set (n = 134). The results in Table 7 show that the method proposed in this paper also demonstrates favorable segmentation performance on the public dataset, indicating good generalization ability.

### 3.6. Confluence Calculation and Cell Counting Effect Analysis of Cellular Images

**Figure 7:**
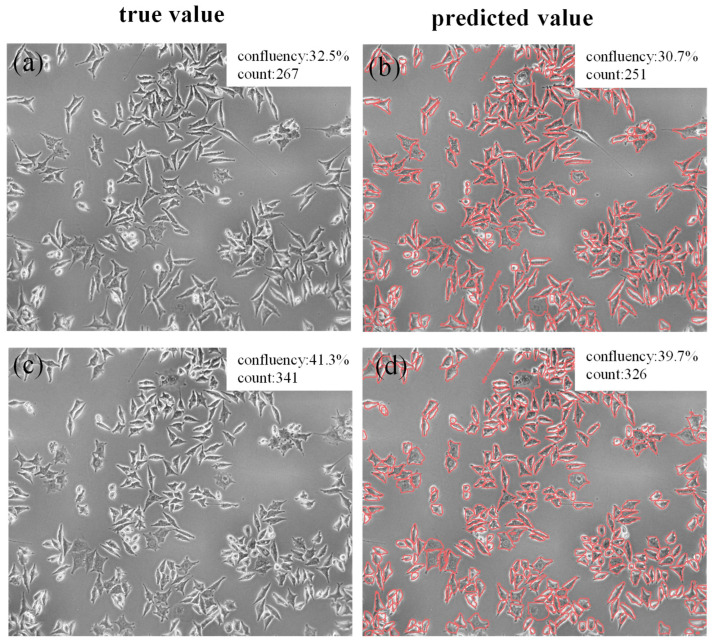
Cell confluence calculation and counting results in GSCs images. (**a**,**c**) The true values of cell confluence and cell quantity; (**b**,**d**) Predicted values of cell confluence and cell quantity.

Cell confluence refers to the ratio of the area covered by cells in a cell culture dish to the total area, while cell number refers to the total number of cells in a cell image. Confluence and cell number are important indicators for evaluating the growth status of cells. To verify the calculation ability of cell confluence and cell number in practical applications, two cell images that were not used for training were selected, as shown in Figure 7a,c. The real confluences were 35.2% and 41.3%, respectively, and the real cell numbers were 267 and 341, respectively. Figure 7b,d shows the results obtained through the method proposed in this paper. The predicted confluences and cell numbers were 30.7%, 251 cells, and 39.7%, as well as 326 cells, respectively. Through comparison, it can be seen that the accuracy of the prediction results is over 94%, indicating that the method can be applied to the real-time analysis of cell confluence and cell number in instruments in the field of biomedical science.

## 4. Discussion

**Figure 8:**
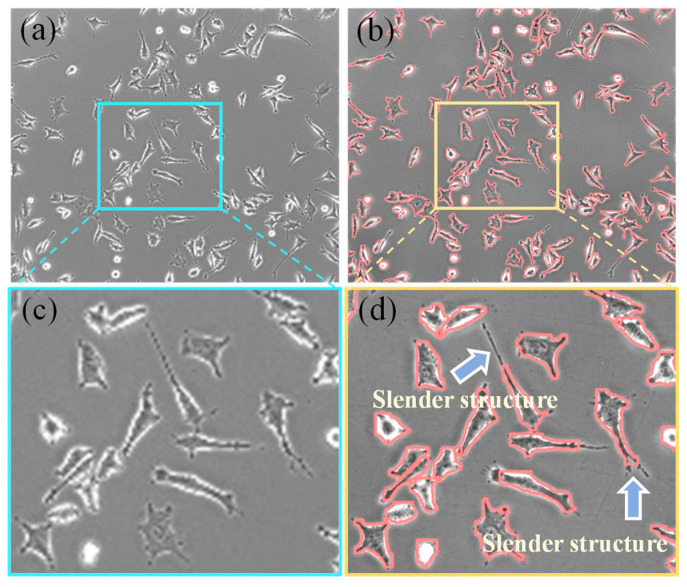
Visual Examples of Incorrect Segmentations: (**a**) Original image, (**b**) Segmentation result, (**c**) Original image detail, (**d**) Segmentation image detail.

In this study, we developed a deep-learning model trained on a novel time-series GSCs dataset, demonstrating high efficiency and accuracy in segmenting highly heterogeneous cells. The integration of BiFPN enhances the fusion of shallow, high-resolution features with deep semantic ones, effectively mitigating the information loss common in standard architectures. This led to notable performance gains, achieving Mask mAP50 and Box mAP50 scores of 0.957 and 0.95, a relative improvement of 3.4% and 3.3% over the baseline, respectively. Concurrently, the AKConv module reduced model parameters by 13.9% while maintaining a real-time inference speed of 38 fps, establishing our framework as a robust analytical tool for GSCs research. The proposed framework presents a powerful alternative to traditional manual analysis, which is often time-consuming and error-prone. By enabling automatic, real-time segmentation, our model provides non-destructive, quantifiable metrics such as cell confluence and counts. These indicators are critical for the continuous spatiotemporal analysis of cellular invasion, holding significant promise for applications in early cancer diagnosis and novel drug development. The model’s strong generalization was further validated by its outstanding performance on two public datasets, confirming its broad applicability. Despite these promising results, our study has limitations that outline clear directions for future work. First, the training data were derived exclusively from brightfield microscopy. Future work will incorporate multi-modal microscopic data to enhance the model’s robustness and generalizability across diverse imaging conditions. Second, as shown in Figure 8, the model tends to produce overly smoothed segmentation boundaries, which can obscure fine cellular details. While this has a minimal impact on quantitative tasks like cell counting, it may limit applications requiring precise morphological analysis. Therefore, we plan to improve boundary definition by incorporating boundary-aware mechanisms or high-resolution refinement branches. Looking ahead, integrating such algorithms into microscopic instruments holds considerable potential. Real-time metrics could directly aid cancer diagnosis and grading, while continuous monitoring of cellular dynamics could accelerate the evaluation of targeted drug sensitivity, thereby advancing both disease diagnosis and drug discovery.

## 5. Conclusions

In this study, we present a novel segmentation framework for GSC images, integrating multi-scale feature enhancement with dynamic receptive field optimization. By introducing a BiFPN, the framework effectively captures fine-grained cellular structures through enhanced multi-scale feature fusion. Additionally, we propose the AKConv module, which dynamically modulates convolutional kernel shapes and sizes, thereby improving the model’s adaptability to the intricate and irregular morphology of glioma cells. Comprehensive experimental evaluations demonstrate that the proposed method substantially advances the precision of GSCs segmentation, supporting applications such as quantification of cell confluence and enumeration, which are critical for investigating physical interactions within the tumor microenvironment. These capabilities hold promise for facilitating disease diagnosis and informing the development of targeted therapeutic strategies.

Future work will focus on validating the generalizability of the model across multi-modal imaging datasets, including fluorescence and electron microscopy. Furthermore, we plan to employ knowledge distillation to compress the model, facilitating its deployment on portable devices for real-time, point-of-care pathological analysis.

## Figures and Tables

**Table 1:** Training parameters used in this study.

Training Parameters	Numeric
Image size	640 × 640
Batch size	16
Epoch	200
SDG momentum	0.9
SDG initial learning rate	0.001
SDG weight decay	0.0005

**Table 2:** Results of ablation experiments using the improved YOLOv8s-seg; the best result is bolded.

Model	Box			Mask			Param (KB)	Weight (MB)	FPS
	Precision	Recall	mAP_50_	Precision	Recall	mAP_50_			
yolov8s-seg	0.934	0.9	0.923	0.933	0.895	0.917	11,780	23.9	13
YOLOv8s-seg + Soft-NMS	0.942	0.91	0.934	0.941	0.903	0.93	11,993	23.9	13.68
YOLOv8s-seg + BiFPN + Soft-NMS	0.953	0.923	0.947	0.95	0.905	0.944	12,035	22.1	19.84
YOLOv8s-seg + BiFPN + Soft-NMS + Akconv	**0.962**	**0.913**	**0.957**	**0.956**	**0.906**	**0.95**	10,364	21.1	38

**Table 3:** The impact of different numbers of layers of Bifpn on the model performance, baseline model is YOLO8s + Soft-NMS.

Number of Layers	Box			Mask			Param (KB)	Weight (MB)	FLOPs (G)	FPS
	Precision	Recall	mAP_50_	Precision	Recall	mAP_50_				
3	0.944	0.899	0.93	0.94	0.891	0.927	10,953	22.2	43.2	22.63
4	0.946	0.904	0.939	0.942	0.893	0.933	10,920	22.2	42.8	22.94
5	0.953	0.923	0.947	0.95	0.905	0.944	10,887	22.1	42.6	19.84

**Table 4:** The impact of convolutional modules in different locations on the model performance, baseline model is YOLOv8s + Soft-NMS.

Location	Box			Mask			Param (KB)	Weight (MB)	FLOPs (G)	FPS
	Precision	Recall	mAP_50_	Precision	Recall	mAP_50_				
Backbone	0.941	0.888	0.93	0.941	0.881	0.925	10,480	21.3	41.9	17
neck	0.943	0.899	0.937	0.942	0.895	0.933	10,561	21.4	42.4	35.34
Backbone + neck	0.883	0.824	0.888	0.877	0.812	0.871	10,487	20.2	40.8	24.04

**Table 5:** Comparison of the segmentation performance of different Yolo models; the best result is bolded.

Model	Box			Mask			Param (KB)	FLOPs (G)	Weight (MB)
	Precision (%)	Recall (%)	mAP_50_ (%)	Precision (%)	Recall (%)	mAP_50_ (%)			
YOLOv3tiny-seg	0.642	0.566	0.615	0.403	0.404	0.345	14,099	32.7	28.4
YOLOv5s-seg	0.903	0.867	0.907	0.894	0.846	0.888	9765	37.8	19.9
YOLOv5n-seg	0.81	0.657	0.754	0.784	0.6	0.702	2755	11.0	**5.8**
YOLOv8s-seg	0.934	0.9	0.923	0.933	0.895	0.917	11,780	42.4	23.9
YOLOv8n-seg	0.936	0.821	0.895	0.932	0.806	0.883	3258	12.0	6.8
YOLOv10n-seg	0.954	0.742	0.852	-	-	-	**2695**	**6.7**	**5.8**
YOLOv10s-seg	0.954	0.75	0.86	-	-	-	8036	21.6	16.5
YOLOv11s-seg	0.934	0.889	0.919	0.925	0.882	0.911	10,067	35.5	20.5
(Ours)	**0.962**	**0.913**	**0.957**	**0.956**	**0.906**	**0.95**	10,364	41.4	21.1

**Table 6:** Comparative performance of cell segmentation with other, different methods; the best result is bolded.

Model	Param (KB)	Box		Mask	
		Precision	mAP_50_	Precision	mAP_50_
Mask R-CNN	43,997	0.776	0.715	0.737	0.691
ConvNeXt-V2	28,676	0.732	0.699	0.697	0.631
SOLO	45,925	-	-	0.698	0.457
SOLOv2	46,299	-	-	0.683	0.564
YOLACT	47,365	0.687	0.592	0.651	0.464
YOLOv8s-seg	11,780	0.934	0.923	0.933	0.917
ASF-YOLO	11,957	0.941	0.923	0.93	0.914
(Ours)	**10,364**	**0.962**	**0.957**	**0.956**	**0.95**

**Table 7:** Comparison of segmentation results of different models on the DBS2018; the best result is bolded.

Model	Box			Mask			Param (KB)	FLOPs (G)
	Precision	Recall	mAP_50_	Precision	Recall	mAP_50_		
YOLOv3tiny-seg	0.924	0.839	0.894	0.792	0.71	0.716	14,099	32.7
YOLOv5s-seg	0.919	0.852	0.922	0.913	0.864	0.906	9765	37.8
YOLOv5n-seg	0.914	0.841	0.898	0.911	0.856	0.899	2755	11.0
YOLOv8s-seg	0.936	0.863	0.919	0.926	0.85	0.91	11,780	42.4
YOLOv8n-seg	0.925	0.843	0.905	0.917	0.831	0.895	3258	12.0
YOLOv10n-seg	0.911	0.853	0.915	-	-	-	**2694**	**6.7**
YOLOv10s-seg	0.922	0.869	0.925	-	-	-	8036	24.4
YOLOv11s-seg	0.937	0.876	0.928	0.932	0.864	0.915	10,067	35.5
YOLOv8s-ASF	0.941	0.891	0.923	0.93	0.863	0.914	11,957	44.3
(Ours)	**0.949**	**0.875**	**0.943**	**0.929**	**0.865**	**0.935**	10,365	41.4

## Data Availability

The raw data supporting the conclusions of this article will be made available by the authors on request. Also, we provide the code of this paper at the following link: https://github.com/LCOUD-ALT/A-real-time-cell-image-segmentation-method-based-on-multi-scale-feature-fusion--code, accessed on 1 January 2025.
